# Association of ergonomics risk factors among Iranian calico crafts-men and musculoskeletal discomforts: a cross-sectional study

**DOI:** 10.1186/s12891-023-06219-x

**Published:** 2023-02-09

**Authors:** Mahnaz Shakerian, Reza Esmaeili, Masoud Rismanchian

**Affiliations:** 1 Department of Occupational Health and Safety Engineering, School of Health, Isfahan University of Medical Sciences, Isfahan, Iran; 2 Student Research Committee, Department of Occupational Health and Safety Engineering, School of Health, Isfahan University of Medical Sciences, Isfahan, Iran

**Keywords:** Calico, CMDQ, Ergonomics, Handicrafts, QEC, Iran

## Abstract

**Background:**

Work-related musculoskeletal disorders (WMSDs) have always been complaints reported by handicraft workers due to the repetitive and static nature of work. Therefore, the current study aimed to investigate the ergonomic conditions of craftsmen engaged with the calico industry.

**Method:**

This cross-sectional study was done in small calico industrial workshops in Isfahan, Iran, in 2022. The sample selection method in this study was census. Using QEC (Quick Exposure Checklist) and Cornell-Musculoskeletal Discomfort Questionnaire (CMDQ), the ergonomic status of workers was evaluated. The data were analyzed using Chi-square (χ2) and Pearson’s product-moment correlation test.

**Results:**

The risk level of working postures in wrist/hand, shoulder /arm, and neck were high or very high among the craftsmen. The relationships between discomfort complaints reported by the participants and the risk level of working postures were significant for neck χ^2^ (1, *N* = 100) = 13.603, *P*_value_ = 0.034, left knee χ^2^ (1, *N* = 100) = 12.310, *P*_value_ = 0.030 and lower left leg χ^2^ (1, *N* = 100) = 11.906, *P*_value_ = 0.042. The posture risk level scores for %57.7 of the participants who self-reported the right shoulder discomfort were found to be high or very high.

**Conclusions:**

The high risk found in most calico craftsmen may induce more serious musculoskeletal problems that require applicable interventions. The most important risk factors realized among calico craftsmen were prolonged sitting postures and repetitive tasks.

## Introduction

The handcrafting sector represents a small industry that produces a variety of valuable items primarily intended to supply the travel and tourism sector. Therefore, as an integral part of the informal economy, crafts generate income, create employment opportunities and thus combat poverty [1]. Also, they play an essential role in economic affairs in many countries due to their major effects on capital and productivity [2]. Handicrafts are expressed as any activity through which useful and decorative products are entirely created by hand or by applying only simple tools with an essential prerequisite of individual skill and creativity [3, 4]. Handcrafting is mostly performed individually in small workshops or home workrooms still active in many developing countries such as Iran, Pakistan, Bangladesh, Turkey, and China, and contains a major part of the workforce. Calico is considered one of the most important handicrafts of Isfahan (the second biggest city in Iran) due to its effects on national capital and job creation (especially in the tourism industry). Calico is an industry through which floral textile is produced using woodblock printing. The printing process is mostly done using certain stamps struck by craftsmen on the clothing surface. This printed clothing has several useful applications, especially in traditional decorations such as curtains, bedcovers, etc. The extended usage of calico, inducing a large number of people to be involved with such an industry, necessitate close health – care attention to these sectors [3, 5, 6].

Handcrafting is a highly decentralized sector where machinery is not dominant and is one of the professions where craftsmen are often exposed to a variety of work-related risk factors, such as prolonged static sitting, strenuous force exertion, and repetitive upper limb movement, all may contribute to work-related health problems [7, 8]. Depending on the work's nature and the hand tools' design, craftsmen may experience awkward postures, strong grips, high repetition frequencies, and the danger of hand-arm vibration (HAV) which can lead to the manual performance disability in them [4, 9]. Researchers have also illustrated many workplace physical and psychosocial risk factors increasing musculoskeletal disorders (MSDs) [10]. These factors include repetitive work, long-lasting sitting static postures, fatigue, job stress, inappropriate tools, workplace, etc. These risk factors induce pain and discomfort in different parts of the body, such as the lower back, neck, shoulders, wrists, and lower limbs [8, 11]. The literature review has offered a wide variety of evidence confirming the negative effects of MSDs on the productivity and health of the workforce all over the world. For example, some studies have shown that the prevalence of manual MSD symptoms among handcrafting occupations ranges from 38.5% to 96.5% [8, 12–14]. Also, statistical studies in the United States in 2006 illustrated that nearly %30 of diseases and injuries inducing at least one working day loss had been associated with MSDs [15]. In addition, a study conducted in -block textile printing industries in India found that the prevalence of MSDs in different body parts was between 15 and 78% [16].

Moreover, Chandni and Neeta surveyed workers employed in such traditional small industries (SSIs) in India to identify ergonomic risks associated with MSDs through assessments using rapid entire body assessment (REBA). They concluded that 92–95% of craftsmen are at risk of MSDs. Also, according to a review study conducted in Iran, the prevalence of MSDs among handicraft workers varied between 38 and 68% [17].

Various studies have been conducted worldwide and in Iran to evaluate MSDs among handicraft workers and ergonomic evaluation of their working environments [7, 18–21]. For example, Rathore et al., in a study in India, surveyed glass art ware workers and its association with MSD symptoms. A detailed analysis of the existing workplaces of artwork workers showed improper workplace design, awkward posture, excessive thermal stress, improper lighting, and inappropriate ventilation, which may create poor working conditions for workers [21]. Also, in Indonesia, Ramdan et al. conducted a study on women weavers working with handlooms to assess factors affecting the prevalence of MSDs among them. They showed that MSD prevalence was significantly correlated with education background, working period, prolonged sitting hours, work posture, and weavers` anthropometric features [20].

Moreover, Das and Singh in India stated that among the studied handicraft workers, most of the participants reported a high prevalence of MSDs, primarily in the neck, lower back, and knee regions. Also, they revealed that age, work experience, prolonged work hours, sustained awkward posture, continuous work without breaks, extensive work pressure, and poor job control increased the risk of MSD symptoms in different body regions [7]. In another study, Dianat et al. and Veisi et al. in two different studies in Iran evaluated working conditions of hand-sewn shoemaking, as a handicraft in Iran, about the incidence of MSDs and found a very high prevalence of musculoskeletal pain in the study population [18, 19]. Considering a wide variety of tasks in different handicrafts with an extensive alteration in their production process, generalizing the results of the previous studies on the handicrafts industry to other similar industries seems nearly illogic. On the other hand, although several studies have been recently carried out on different types of handicraft workers, particularly carpet weaving in Iran, many other crafts, including the calico industry, require ergonomically investigating and redesigning. The present study, therefore, was performed to evaluate the ergonomic status of craftsmen in the calico industry using the QEC method and CMDQ.

## Methodology

### Participants

The present study population consisted of calico industry craftsmen who were all male in Isfahan (the second biggest city in Iran). The sampling method used to select participants in this study was census; all workers with at least one year of working experience were included. Therefore, all workers involved with the calico industry (100 craftsmen) participated in this study, working in 25 workshops, each with an almost capacity of 4 to 5 workers. Also, most participants were right-handed (98% of the total population). Before the study, a written informed consent form was given to each participant and an opportunity to refuse to attend this study. Participants were initially informed about the study purpose in a familiarization session outlining all study steps. In the current study, participants were selected based on the following recruitment criteria:

a: No cardiovascular diseases; b: Having mental health with no mental disorders; c: No use of caffeinated beverages regularly; d: No use of hypnotic drugs; e: No drug addiction and smoking; f No sensible musculoskeletal diseases (e.g., carpal tunnel syndrome (CTS), low back pain); g: No sleep disorders; h: No major systemic diseases; i: Having more than one-year working experience.

### Study design

**Fig. 1:**
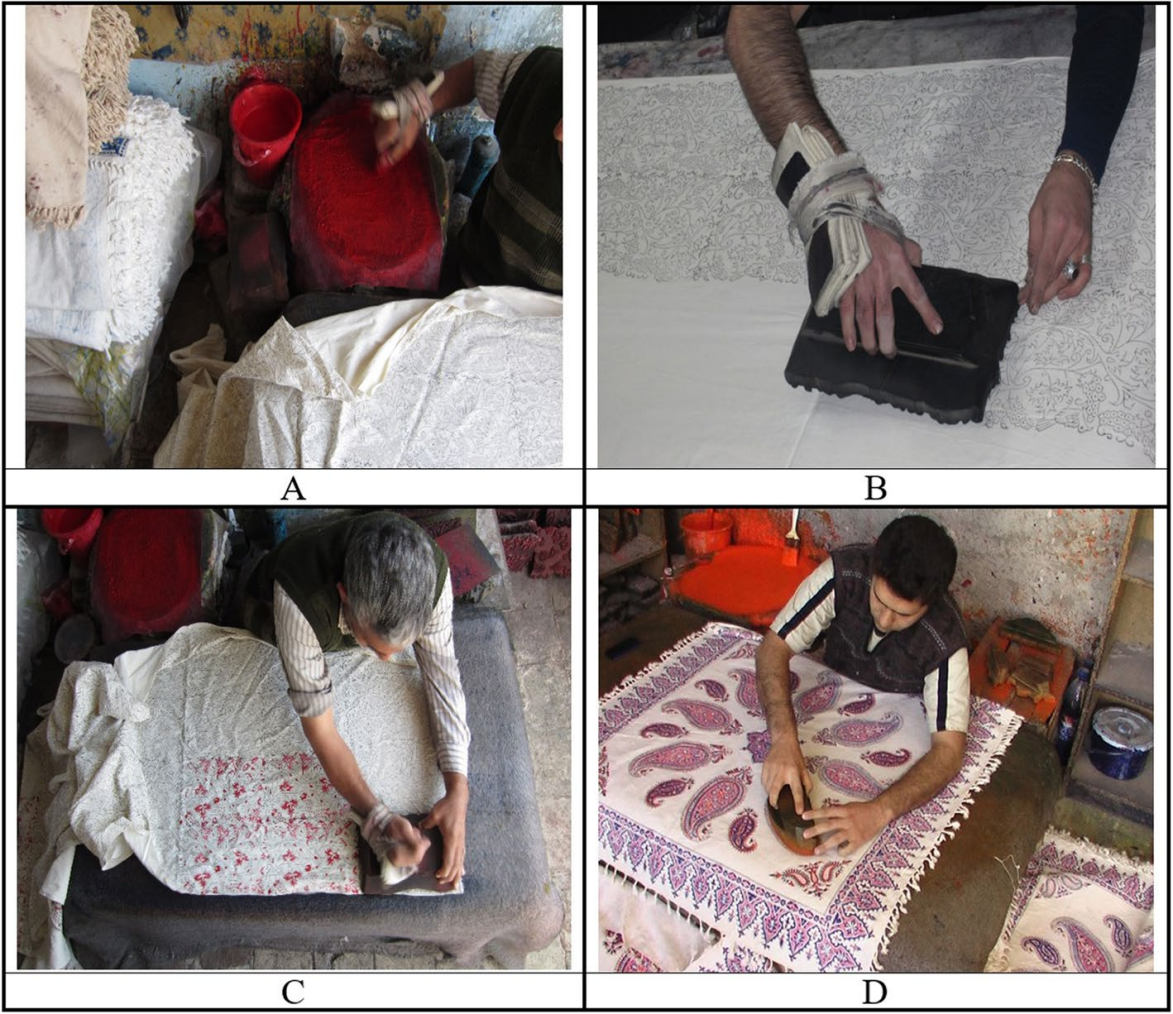
Different steps in the crafting process; **A**: Color making; **B**: Mapping; **C**: Primary coloring; **D**: Final coloring

This study was a cross-sectional study with an analytical-descriptive approach. Data were collected using direct observation of the working process, performed by the research team, and a questionnaire. The present study was a part of the output of a project related to the handicraft industry, which was carried out in four months from April to August 2022, when these workshops were at their peak production rate. Before the study, to obtain more desirable results, all work positions in the calico industry, including those workers involved with the fabric printing process, were precisely analyzed through observations by the research team. The observation took place in all workshops directly or by recording videos using a closed-circuit television (CCTV) camera. Finally, the present study was conducted in 3 main stages: 1. Collecting participants' demographic information; 2. Evaluation of MSDs among the craftsmen using CMDQ; 3. Assessment of participants' working posture using quick exposure check (QEC) technique. Figure 1 shows different steps in the crafting process.

#### Demographic information of the participants

A form was prepared by the research team. It was arranged basically concerning the demographic information, including the participants´ age, height, weight, body mass index (BMI), educational level (illiterate, primary school, secondary school, diploma, and BA, and more) and marital status (single or married). Some required individual habits, such as smoking habits and the amount of physical activity, were also considered. The information about their job, including daily working hours, job experience (years), and the number of hours they work without any break (how many hours do you work without any break?), were all collected through visiting sessions by the research team and were recorded in demographic forms.

#### Cornell Musculoskeletal Discomfort Questionnaire (CMDQ)

The CMDQ was advanced by Hedge et al. [22] and was adapted in Persian by Affifzadeh et al., through which its validity and reliability were evaluated, and the Cronbach's alpha coefficient was 0.986 [23]. CMDQ is a three-part survey that includes body map charts and the prevalence of musculoskeletal pain or discomfort in 20 areas (neck, lower back, back, etc.) during the last week. At the first part of the questionnaire, workers' responses are graded from "never = 0 points" to "several times a day = 4 points". Those who select “never” in this part will not require completing the other two parts. The second part examines the pain intensity of the respondents and rates their responses from " slightly = 1 point" to "very = 3 points". The third part assesses whether pain interferes with their work, and the answer is graded between "no at all = 0 points" and "very = 2 points". Regarding the final score of the questionnaire, achieving a higher score means higher discomfort frequency, severity, and disability [24]. In general, to assess the prevalence of MSDs in industries, one of two tools, the CMDQ or the Nordic questionnaire [25], is usually used. Considering that the CMDQ provides researchers with complete information on the scoring of pain in different body parts, the CMDQ was also used in the current study. In this study, the areas of the body to be evaluated included low back, shoulder/forearm, wrist, and neck. Moreover, regarding the observation and the craftsmen reports, some required data, including the maximum weight of the handled object, the maximum exerted force with one or both hands, the exposure to vibration factor while working, and visual factors with high accuracy required, were to be collected. According to the study intent, CMDQ was used to evaluate the presence of musculoskeletal pain, discomfort, or disorder, the intensity of the discomfort feeling, and the possible effects on the workability of the craftsmen while working. To obtain more exact results, the participants were instructed on how to fill out the questionnaires before the beginning of the study. To meet the study intent, the research team attended target workshops when completing the questionnaires for the participants.

#### Quick Exposure Checklist (QEC) method

The quick exposure checklist (QEC) is considered a common observation method developed in the United Kingdom between 1996 and 1998. This method assesses—WMSDs risk factors that affect the upper body limbs and also rates exposure to vibration and work stress. The QEC method was specifically designed to meet the needs of ergonomists and professionals by involving both observers and workers in assessing work tasks [26]. QEC was designed to assess the risk of a single task item at a time. The evaluation is performed in two sections. The evaluator assesses the craftsman's working posture and back, shoulder/arm, wrist/hand, and neck movements in the first section. In the second section, the evaluator is concerned about the weight processed during work, the strength of the hands, the time spent per day on the work, the visual demands of the work, the driving of the vehicle, the use of vibrating tools, and the difficulty of continuing the work and work stress. The QEC instructions state that the worst-case situation needs to be assessed, for example, when your elbows are the most bent. A total score is given for each body part, and the scores are grouped into four exposure levels (low, medium, high, and very high) [27]. In the QEC method, there is a checklist/score sheet to evaluate and collect the necessary information, which can be completed by the observer and the operator. So, this method allows the researcher to evaluate the worker's subjective opinion on how the work is done. In addition, this technique allows the evaluation of tasks such as manual carrying of loads, repetitive tasks, static tasks, dynamic tasks, and sitting and standing tasks, all of which are under the tasks performed by the craftsmen [27]. Accordingly, this method was used to evaluate the participants' posture in the recent study. In the present study, the independent variables were the participants' ergonomics status, including awkward postures measurable through QEC. The postures in the calico process were distinguished through several observation events performed by the research team. The postures were found as long as workers´ daily hours for the majority of the participants, and work-related psychological stressors were comprehensively recorded for those jobs with repetitive activities. Initially, 20 to 30 cycles of a certain job are recommended to be observed. It is noteworthy that the remarkable feature of QEC to be used is the cooperation and interaction found between the observers and the participants while working in all different evaluation steps. Initially, the priority to be investigated is selected. Then, the researcher observes workers’ posture accurately while asking certain questions to the participants about the posture status and different parts of the body movements. Then, the other required questions in the checklist would be answered by the participant.

### Data analysis

SPSS_19_ (SPSS Inc., Chicago, IL, USA), was used for all statistical analysis. In the current research, the analyzed data had a normal distribution. The statistical tests used in this study included the chi-square test to evaluate qualitative data. Pearson's product-moment correlation was used to determine correlations between MSDs and independent variables (age, education, work experience, BMI, average daily working). A normality test was performed before the analysis. The significance level in this study was less than 0.05 (*P*-value ≤ 0.05).

## Results

### Demographic information and iob details

All healthy male craftsmen involved with calico printing were recruited for the study from Isfahan, Iran. Male participants were selected to be evaluated in this study as most of the calico industry population was men. Participants had a mean age of 33.16 years (SD =  ± 10.64, range = 16 to 58). Also, %70 of the participants reported 8 to 10 daily work hours involving calico fabric printing, and %30 of them reported experiencing daily working hours in this process for at least 10–12 h. Moreover, %41 of the participants reported experience working in the calico industry for 1 to 10 years. Furthermore, the majority of the participants (%58) had a normal BMI (body mass index) the range of 18.5 to 25. Table 1 shows the demographic information of participants in the current study.

**Table 1:** demographic information of participants

Variable	Status	Number (Percentage)
Marital status	Single	33 (33%)
	Married	67 (67%)
Education	Illiterate	5 (28%)
	Primary school	28 (28%)
	Secondary school	33 (33%)
	Diploma	31(31%)
	Associate’s degree	0
	Bachelor and upper	3(3%)
Work experience (year)	< 10	41(41%)
	10–20	38(38%)
	> 20	21(21%)
BMI	< 18.5	6(6%)
	18.5–24.9	58(58%)
	25–29.9	29(29%)
	30–34.9	7(7%)
	> 35	0

### Self-reported musculoskeletal symptoms

According to the extracted results of CMDQ, the highest percentages of sever discomfort complaints were related to the right shoulder (%36), right wrist (%26), neck (%25), and upper right arm (%24), respectively. Moreover, based on CMDQ, %45 of the participants reported that pain and discomfort in the right shoulder had highly affected their working ability. The highest percentages related to the effects of discomfort feeling on the ability to work after the right shoulder were found in the right wrist (%34), neck (%25), upper right arm (%24), and lower back (%24), respectively. Table 2 represents the results related to the distribution of musculoskeletal discomforts and their impacts on workability of calico craftsmen.

**Table 2:** Distribution of musculoskeletal discomfort and pain impact on the ability of the participants

Regions		The pain of the working people (%)			The impact of pain on activity (%)		
		Low	moderate	high	Low	moderate	high
Neck		11	30	25	3	38	25
Shoulder	Right	7	33	36	3	28	45
	Left	3	14	5	3	11	8
Upper Back		11	29	12	3	31	18
Lower Back		13	27	17	5	28	24
Upper arm	Right	20	21	24	9	32	24
	Left	5	13	5	2	14	7
Forearm	Right	7	20	18	5	17	23
	Left	8	8	1	4	11	2
Wrist	Right	15	22	26	4	25	34
	Left	6	4	1	2	7	2
Hips		17	19	5	8	25	8
Thigh	Right	9	18	6	2	18	13
	Left	8	12	3	2	13	8
Knee	Right	14	18	14	8	21	17
	Left	8	12	12	7	13	12
Lower leg	Right	10	17	6	2	22	9
	Left	8	12	1	2	15	4

Table 3 illustrates the relationship between some demographic characteristics, including age, BMI, mean working experience years, mean daily working hours, and the occurrence of feeling discomfort or pain reported in more involved musculoskeletal areas of the body (neck, upper right arm, low back, right forearm) among the participants. The results indicated that the relationship between mean working years and reported musculoskeletal discomfort among the involved areas, including the upper right arm and low back, had not been statistically significant. However, the relationship between BMI and neck discomfort was significant (*r *= 0.672, *P* value = 0.023). Also results showed that the relationship between Working Experience years and Low back discomfort was significant (*r* = 0.538, *P* value = 00.35).

**Table 3:** The relationship between demographic characteristics and the frequency of musculoskeletal disorders among the participants using pearson's product-moment correlation

**Variable**	**Mean ± SD**	Neck		Upper Arm Right		Low back		Forearm Right	
		*r*	*P*	*r*	*P*	*r*	*P*	*r*	*P*
Age	33.16 ± 10.64	0.257	0.205	0.137	0.402	0.102	0.572	0.072	0.860
BMI	23.97 ± 3.68	0.672	0.023^*^	0.105	0.397	0.065	0.988	0.219	0.281
Working Experience years	15.9 ± 11.08	0.302	0.079	0.147	0.163	0.538	0.035^*^	0.239	0.221
Average daily working hours	10.27 ± 1.28	0.195	0.180	0.086	0.698	0.137	0.350	0.078	0.857

^*^ Significance level was considered as *p*-value < 0.05

### Exposure levels (based on QEC method)

Based on the study objectives, the risk level of working postures related to the involved areas among the participants was evaluated using the QEC method. The risk level evaluation results for the calico craftsmen's neck posture were found to be very high for %24 and moderate for %58 of the participants. The QEC risk level score calculated for the region of the shoulder/arm illustrated a high-risk level for %29 and a moderate risk level for %30 of the participants. Furthermore, based on the QEC score, %31 of the participants indicated a high or very high-risk level for the posture of the wrist/hand. Risk level evaluation in four main regions of the body is represented in Table 4, based on the calculated score of the participants.

**Table 4:** Assessment of exposure (risk levels) in four regions among the participants

Region Rating	Low (20–10)	Medium (30–21)	High (40–31)	Very high (56–41)
Low back	30 (30%)	53 (53%)	9 (9%)	8 (8%)
Shoulder / Arm	33 (33%)	30 (30%)	29 (29%)	8 (8%)
Wrist /Hand	26 (26%)	43 (43%)	24 (24%)	7 (7%)
Neck	5 (5%)	58 (58%)	13 (13%)	24 (24%)

### Frequency of musculoskeletal disorders in different regions of the body

Table 5 represents the frequency of reported musculoskeletal discomforts in different body regions based on working posture, and risk levels, and the relationship between these variables using the chi-square test. Considering the results, the risk level of working postures and reported musculoskeletal discomfort in the neck, right shoulder, upper right arm, left knee, and lower left leg were related but not statistically significant for neck χ2 (1, *N* = 100) = 13.603, *P* value = 0.034, left knee χ2 (1, *N* = 100) = 12.310, *P* value = 0.030 and lower left leg χ2 (1, *N* = 100) = 11.906, *P* value = 0.042.

**Table 5:** Frequency of musculoskeletal disorders in different regions by the level of risk posture using the chi-square test

The musculoskeletal Region	level of risk posture				df	Chi- square	*P*-value
	**Low Number (%)**	**Moderate Number (%)**	**High Number (%)**	**Very high Number (%)**			
Neck	18(27.3)	9(13.6)	31(47)	8(12.1)	1	13.603	0.034 *
Right Shoulder	18(23.7)	14(18.4)	35(46.1)	9(11.8)	1	10.259	0.114
Left Shoulder	4(18.4)	5(22.7)	11(50)	2(9.1)	1	3.263	0.775
Upper back	12(23.1)	8(15.4)	24(46.3)	8(15.4)	1	5.028	0.540
Right Upper Arm	15(23.1)	12(18.5)	30(46.2)	8(12.3)	1	6.985	0.322
Left Upper Arm	5(21.7)	3(13)	11(47.8)	4(17.4)	1	1.213	0.976
Lower back	14(24.6)	9(15.8)	25(43.9)	9(15.8)	1	9.522	0.146
Right Forearm	11(24.4)	7 (15.6)	22(48.9)	5 (11.1)	1	6.514	0.368
Left Forearm	5(29.4)	3(17.6)	6 (35.3)	3(17.6)	1	9.067	0.170
Right Wrist	15(23.8)	13 (20.6)	27 (42.9)	8 (12.7)	1	5.337	0.501
Left Wrist	2 (18.2)	2 (18.2)	4 (36.4)	3 (27.3)	1	6.799	0.340
Hips	11(26.8)	5 (12.2)	21 (51.2)	4(9.8)	1	5.615	0.468
Right Thigh	9 (27.3)	4 (12.1)	16 (48.5)	4 (12.1)	1	1.120	0.981
Left Thigh	8 (34.8)	3 (13)	11(47.8)	1 (4.3)	1	11.112	0.085
Right Knee	10 (21.7)	7 (15.2)	24 (25.2)	5 (10.9)	1	9. 805	0.133
Left Knee	7 (21.9)	6 (18.8)	17(53.1)	2 (6.3)	1	12.310	0.030 *
Right Lower leg	10(30.3)	5 (15.2)	14(42.4)	4(12.1)	1	1.952	0.924
Left Lower leg	7 (33.3)	4 (19%)	8(38.1)	2(9.5)	1	11.906	0.042 *

^*^ Significance level was considered as *p*-value < 0.05

Moreover, the calculated posture risk level scores were found to be high or very high for %57.9 of those participants who had reported right shoulder pain or discomfort. In addition, %58.5 of the participants with experience of upper right arm pain had a high working posture risk level. There was no significant relationship between reported musculoskeletal discomfort of other body regions and working posture risk level (Table 4).

### Relationship between the maximum handled, maximum force, and visual demands with musculoskeletal discomfort

In addition, based on the QEC method, the relationship between other related factors, including the maximum weight of the handled object, the maximum force exertion, visual demands, and reported musculoskeletal discomfort in more involved regions, were investigated among the participants. The results indicated significant relationships between the maximum forces exerted by the participants and reported musculoskeletal discomfort in the upper right arm (*r* = 0.502, *P*_value_ = 0.041) and right forearm (*r *= 0.496, *P*_value_ = 0.047). However, a significant relationship was found between the maximum weight of the handled object and visual demands and reported musculoskeletal among involved regions (neck, upper right arm, low back, and right forearm) (Table 6).

**Table 6:** The relationship between the maximum handled, maximum force and visual demands with musculoskeletal discomfort using pearson's product-moment correlation

Variable	Neck		Right upper arm		Low back		Right Forearm	
	*r*	*p*	*r*	*p*	*r*	*p*	*r*	*p*
Maximum weight handled	0.182	0.411	0.092	0.608	0.190	0.439	0.071	0.815
maximum force level	0.157	0.470	0.502	0.041^*^	0.202	0.309	0.496	0.047^*^
visual demands	0.076	0.805	0.144	0.345	0.238	0.286	0.063	0.904

^*^ Significance level was considered as *p*-value < 0.05

### Evaluation of exposure to vibration, work rate, and stress

The other factors in QEC were work rate and stress, to be evaluated in this study. According to the results, the reported work rate was high for 36% and moderated for 26% of the participants. Furthermore, the reported stress of 38% of the participants was very high or high, and 24% of them claimed moderate stress experience (Table 7). Finally, the total QEC score was calculated and illustrated a very high or high level for 53% of the participants, where implementing the further study and immediate corrective actions seemed highly required.

**Table 7:** Evaluation of exposure to vibration, work rate, and stress

Score Risk Factors	Low	Moderate	High	Very high
Vibration	––-	56%	28%	16%
Work rate	38%	26%	36%	––-
Stress	38%	24%	23%	15%

## Discussion

The present study findings demonstrated a high risk of musculoskeletal discomforts and their effect on the ability to work among calico industry craftsmen. Based on the findings, 36% of craftsmen (nearly 0.3 of the target population) claimed severe pain in the right shoulder. Furthermore, the information extracted from CMDQ illustrated that after the right shoulder, the highest percentages of severe musculoskeletal pain among calico craftsmen referred to the right wrist, neck, and upper right arm, respectively. According to the observations, the most essential part of the calico process regarding the ergonomics approach was printing different patterns on clothes using a variety of wooden stamps in terms of their pattern and size. As the printing task imposes repetitive movements to the involved hand (right hand in %98 of the population), the highest percentages of self-reported complaints of upper limb musculoskeletal pains could be predictable.

Moreover, the printing process requires the worker to strike the stamp using his hand while occasionally turning to the side (right side for %98 of the craftsmen) where the dye plate is placed to provide contact between the wooden stamp and dye. These repetitive turnings toward the dye plate and the hits on the stamps, impose several repetitive movements on the craftsmen and induce the feeling of musculoskeletal discomfort in some regions, such as the right shoulder, right wrist, neck, lower back, and upper right arm. In addition, according to the direct observations performed by the research team and the analysis of videos recorded during the fabric printing, the number of hits by workers´ hands on the stamps would sometimes reach 25 to 30 hits per minute. It is noteworthy that the blocks used in the calico industry were made of a particular wood with heavy weight, so the heaviest block weighed by researchers was nearly 1200gr (the lightest block weighed 200gr). So, compared with other parts of the musculoskeletal system, the high frequency of pain in the shoulder and upper right arm could be due to the repetitive heavy blocks by craftsmen. These findings agree with the previous studies where an increasing trend in WMSDs was observed in the repetitive nature of the jobs [19, 28, 29].

According to the CMDQ findings, the ability to work for %45 of the craftsmen (nearly half of the total participants) was likely to be highly decreased by right shoulder pain. The present study results on the feeling of disability at work due to musculoskeletal discomforts or pains conformed to Meena et al.'s findings [30].

Another item evaluated in the current study was the possible relationship between the demographic characteristics of the participants and the frequency distribution of musculoskeletal discomfort complaints. Achieving a higher χ^2^ when making the relationship between the working years of the craftsmen on average and reported musculoskeletal discomfort in the neck and upper right arm can clarify a higher probability for upper limbs to be engaged with more serious problems. The relationships, though, were not statistically significant. On the other hand, the extracted results from CMDQ showed that %60 of the studied craftsmen had working years of more than 10, and one-fourth of the participants had been more than 20 years working. Because the calico craftsmen mostly spend a considerable number of hours (average 10 h) during a day printing the fabrics. It is more likely to find relationships between working years and the reported complaints of involved musculoskeletal areas. Based on the findings, a relationship can be found between lower back discomfort among the participants and mean working years. In the study of Ramdan et al., performed on women weavers working with handlooms, a significant relationship was found between MSDs and work experience [20]. As the statistical aspects for the age have been considered in this study, this relationship might be due to the nature of calico working that includes some static postures like long–lasting sitting and often repetitive turnings when printing on fabrics by the craftsmen. The lower back pain reported by the majority of working people seems possible in the target handicraft industry. In this regard, also in the study of Ramadan et al., no significant relationship was found between mean age and the prevalence of MSDs [20]. However, in the study of Das et al., the relationship between age and the prevalence of MSDs was significant [31].

Many previous studies have always emphasized the relationship between reported pain or discomfort in the lumbar area and working years among the workers involved with awkward postures and repetitive tasks [3, 8]. The present study results agree with Das et al. in 2018 on observing a relationship between the mean working years for workers involved with the handicraft industries and the number of lower back discomfort reports among them [8]. Moreover, the QEC results followed by the exact evaluation of the posture risk level of each workstation, illustrate a high or very high risk level in the neck for %37 of the study population. It is noteworthy that the printing fabric process requires much preciseness due to the nature of the calico through which a wide variety of patterns with different sizes should be stamped by workers´ hands. Therefore, the craftsmen in calico process must bend over the workbench in an awkward posture to do the task accurately. Interestingly, only %5 of the participants had low-risk level for the neck. Also, a significant statistical relationship was found as a confirmative explanation between neck discomfort feelings reported by craftsmen and working postural risk level. Similar results also were extracted from the studies carried out by Das and Singh in 2021 and Meena et al. in 2012 [7, 30].

Apart from the high-risk level observed for the neck, more than one-third of the craftsmen under the study showed high-risk level for the shoulder/arm region, and %29 of them had a high or very high-risk level in the hand/wrist region, which may reveal the presence of awkward postures in workstations of calico industry and determines the necessity of ergonomically corrective actions for the studied calico craftsmen. It is noteworthy that one of the important deficits refers to not using appropriate back support when long time sitting. In a study done by Didar Hossain et al. in 2018 on evaluating the ergonomics status of Indian workers with similar working postures to those of the calico craftsmen using the QEC method, immediate corrective actions were also considered essential [32].

The current study results also indicate significant relationships between feeling discomfort in the left knee (*p*_value_ = 0.034) and the left lower leg with the risk level of working posture. The research team observations and the videos recorded from the calico process demonstrated the workers' long–term sittings and non-ergonomic situations when fabric printing. The lack of appropriate human factor conditions in the workplace regarding inadequate leg space or permanent non-dynamic postures makes these conditions more intolerable. Noticeably, one assumed reason is repeatedly turnings toward the side where the painting vessel is located (the right side in the majority of the population) that induce craftsmen’s right leg to be more dynamic than the left resulting in a more reported awkward posture for the left leg. At the same time, not using a suitable workbench and chair and just digging a hole under the bench for the legs requires implementing engineering designs with ergonomics principles as an important priority. In the studies performed by Dianat et al. and Veisi et al. on a group of hand-sewn shoe workers, a high prevalence of reported knee pain was found due to inappropriate conditions of sitting on the floor (19, 20). Moreover, in another study by Meena et al. on handicraft workers, the working postures were significantly improved after designing an appropriate workstation [33].

Nevertheless, although a high QEC score was achieved for the risk level of working posture in the right knee and right lower leg, the relationship between the risk level of these regions and reported musculoskeletal discomfort among the participants was not statistically significant. These findings, however, agreed with the obtained results of previous studies [34]. Furthermore, there was no significant relationship between other musculoskeletal regions of the craftsmen and the working posture risk level, which could be challenging considering that some regions of workers’ bodies, such as right wrists and right arms, were highly involved in calico. According to the research team observations, a subtle non-symmetry between right and left arms could be seen among some craftsmen´s arms that may reach 2 to 2.5 cm in some cases. The dissymmetry arms might be due to a mechanical adjustment caused by awkward posture inducing a thicker right arm, particularly among craftsmen with longer working years. In the process of fabric printing in calico, there is a tool named “Moshteh” attached to the involved hand of craftsmen and applied to transfer the force of workers' arms to the fabric stamps equally when printing. Using such a device for a long time must be the reason for the deformity mentioned. In this regard, Antwi-Afar et al., in a study, examined the effect of lifting weights and postures on muscle activity and fatigue among construction workers. Their study showed that lifting weight and postures had a significant relationship with muscle activity and fatigue, which in the long run can lead to muscle deformity [35].

According to the study aims, out of other factors affecting the ergonomic status of calico craftsmen, including maximum weight handled, maximum force exertion level, and visual demands, there were significant relationships between the maximum force exertion level and discomfort feeling in the upper right arm (*r* = 0.502, *P*_value_ = 0.041) and right arm (*r* = 0.496, *P*_value_ = 0.047). It means that feeling ache, pain, or discomfort has been reported more among those craftsmen with higher force exertion when performing the printing process using different wooden blocks. Moreover, discomfort is more likely to be experienced in regions like the right shoulder and right upper arm joints, where the block weights are directly imposed. Notably, the designs assigned for calico clothing mostly change based on market demand. Therefore, the pattern variety requires a wide range of blocks with different sizes and weights. However, based on the craftsmen's claims, the force exertion required by each pattern is not equal and depends on some other factors. For instance, as the concentration and viscosity of all painting colors applied in the calico are not the same, higher force exertion to the stamps could unfavorably cause blurred fabric patterns and may lessen the quality of the products. According to interviewees, some colors, like blue and red, need less force exertion compared to black. Therefore, several factors, such as craftsmen experience, applied patterns, block sizes, and color concentration, might affect the required force exertion. So, making a fair judgment here requires more precise studies. The present study results are parallel with the findings of the previous studies through which force exertion was found as a risk factor for WMSDs [18, 36].

In the present study, a significant relationship existed between BMI and the frequency of MSDs in the neck region (*r* = 0.672, *P* value = 0.023). Considering that participants with higher BMI have lower physical activity and sports activities than those with lower BMI, this lower physical activity can lead to an increase in MSDs in different areas of the body, including the neck. In other words, people who attend regular and planned exercise and physical activity outside the work environment experience less musculoskeletal discomfort caused by work. These results were completely consistent with the study by Elvis et al., who found a significant relationship between neck pain and BMI among the studied welders [37].

### Implications for ergonomic improvements

Finally, the obtained high (%42) or very high (%11) total QEC score of risk level in calico craftsmen demonstrates unfavorable ergonomic conditions which will probably lead to more serious deficiencies if continued and therefore urgent corrective measures are required. The current study also found relatively high values for working speed and stress among the participants. As it was mentioned before, %62 (more than half) of the participants reported high or moderate work speed. This finding can be due to the number of hand hits on the block during a certain time, so the research team estimated 25–30 hits per minute (12,000 to 14,500 in 8 h). Based on the craftsmen claims, the high work rate in the calico industry may be due to high market demand or fear of less productivity. As was observed in the results, %38 of calico craftsmen had high or very high stress. In previous studies on the handicraft industry, nearly similar findings have been extracted [38]. Also, the study by Jadhav et al. on 105 artisans of Kolhapuri crafted footwear manufacturing in India stated that awkward postures inducing WMSDs were reported as the most important problem among the target population [37]. Based on this, it seems that control measures and ergonomics implications such as improving the workstations of these artisans, redesigning the hand tools used by them, reducing their working time, and reducing their work speed can help to reduce musculoskeletal disorders and improving ergonomic condition in them.

### Limitations and recommendations for future research

Nevertheless, the present study also had some limitations. The most crucial point to be expressed is that our study was carried out in one of the rural areas located in Isfahan. Despite using the census as a sampling method, some other expanding studies with more target population are required to make a more precise judgment on the ergonomics conditions of calico industry workers and to generalize the results associated with the relationship between the risk level of working posture and reported musculoskeletal discomforts among calico craftsmen. In addition, it isn't easy to compare our findings with those of the other studies due to the innate calico industry found in some parts of developing countries like Iran. Therefore, generalization of the results to the total population of calico craftsmen seems difficult.

Furthermore, the participants in this study were all men, and generalizing the results to the women's handicraft society involved with calico may be done with some considerations. Another limitation to be mentioned is the subjective information related to musculoskeletal discomforts collected from CMDQ, a self-reported questionnaire. Therefore, the present study could be affected by interfering factors, such as different interpretations of participants that seem inseparable parts of self-reporting surveys. So, designing a cohort is recommended to obtain more reliable results. Finally, based on the current study results indicating a high prevalence of musculoskeletal discomforts and high-risk level of working posture implementing different corrective actions with a certain plan in the target industry is required. Some corrective interventions, including increasing the number of craftsmen, limiting working hours, redesigning workplace layouts such as different wooden blocks or painting vessels, and redesigning the workplace with engineering attention to consider appropriate spaces for different parts of craftsmen's bodies, particularly legs, are recommended. Furthermore, as a recommendation, presenting an appropriate education program, including considering short breaks during working time, correcting awkward postures, and doing some light, regular physical activities for the people involved with calico, could be a feasible solution.

## Conclusion

There is likely a relationship between discomfort among calico craftsmen and its effect on their workability, particularly in the fabric printing process, where involved musculoskeletal regions are affected, and the risk level of working posture and the amount of force exertion during work are relatively high. Achieving a high score for risk level in QEC for the majority of the target population, it can be inferred that calico is considered a high – risk job inducing serious musculoskeletal problems if immediate corrective actions are not implemented.

## Data Availability

Due to the request of the participants in the study and the protection of their privacy, we are exempt from disclosing their personal information publicly. The datasets used and analyzed during the current study are available from the corresponding author on reasonable request.
